# OsRELA Regulates Leaf Inclination by Repressing the Transcriptional Activity of OsLIC in Rice

**DOI:** 10.3389/fpls.2021.760041

**Published:** 2021-10-01

**Authors:** Chen-li Zhu, Bao Xing, Shou-zhen Teng, Chen Deng, Zhen-yong Shen, Peng-fei Ai, Tie-gang Lu, Sheng-wei Zhang, Zhi-guo Zhang

**Affiliations:** ^1^Joint CAAS/IRRI Laboratory for Photosynthetic Enhancement, Biotechnology Research Institute/National Key Facility for Genetic Resources and Gene Improvement, Chinese Academy of Agricultural Sciences, Beijing, China; ^2^Ministry of Education Key Laboratory of Molecular and Cellular Biology, Hebei Collaboration Innovation Center for Cell Signaling and Environmental Adaptation, Hebei Key Laboratory of Molecular and Cellular Biology, College of Life Sciences, Hebei Normal University, Shijiazhuang, China; ^3^College of Bioscience and Bioengineering, Hebei University of Science and Technology, Shijiazhuang, China

**Keywords:** leaf inclination, *OsRELA*, *OsLIC*, phytohormone, rice

## Abstract

Leaf angle is one of the most important agronomic traits in rice, and changes in leaf angle can alter plant architecture to affect photosynthetic efficiency and thus determine grain yield. Therefore, it is important to identify key genes controlling leaf angle and elucidate the molecular mechanisms to improve rice yield. We obtained a mutant *rela (regulator of leaf angle)* with reduced leaf angle in rice by EMS mutagenesis, and map-based cloning revealed that *OsRELA* encodes a protein of unknown function. Coincidentally, *DENSE AND ERECT PANICLE 2* (*DEP2*) was reported in a previous study with the same gene locus. *RNA-seq* analysis revealed that *OsRELA* is involved in regulating the expression of *ILI* and *Expansin* family genes. Biochemical and genetic analyses revealed that OsRELA is able to interact with OsLIC, a negative regulator of BR signaling, through its conserved C-terminal domain, which is essential for OsRELA function in rice. The binding of OsRELA can activate the expression of downstream genes repressed by OsLIC, such as *OsILI1*, a positive regulator of leaf inclination in rice. Therefore, our results suggest that OsRELA can act as a transcriptional regulator and is involved in the regulation of leaf inclination by regulating the transcriptional activity of OsLIC.

## Introduction

As one of the agronomic traits closely related to grain yield, leaf angle not only controls plant architecture but also moderate leaf erectness is essential for improving rice yield (Wang and Li, 2011). Leaf erectness improves light capture and CO_2_ diffusion efficiency (Sakamoto et al., 2006), thus increasing photosynthetic efficiency. The leaf angle in rice is determined by the lamina joint that connects the leaf blade and leaf sheath. Compared with the leaf tissue, mesophyll cells were replaced by parenchyma cells, and more sclerenchyma cells were gathered around the vascular bundles on the adaxial and abaxial sides. This structure not only provides mechanical strength to the lamina joint but also a determinant factor of leaf angle (Zhou et al., 2017). Many genes have been reported to be involved in the regulation of lamina joint development in rice, and many of these mutants have shown great potential to increase yield. Therefore, it has become the goal of breeders to find and elucidate the molecular mechanism of genes in regulating leaf angle in rice.

In rice, many transcription factors have been reported to be involved in the regulation of lamina joint development; therefore, control of leaf angle can be achieved through genetic manipulation. Altering the expression of some specific transcription factors resulted in increased leaf angle; for example, overexpression of the *Lax panicle1 (LAX1)* gene resulted in increased leaf angle (Komatsu et al., 2003), while the *OsLIC (LEAF and TILLER ANGLE INCREASED CONTROLLER)* mutant exhibited a significant increase in leaf angle and tiller angle (Wang et al., 2008). Overexpression of *auxin response Factor 19* (*OsARF19*) can promote cell division on the adaxial side of the lamina joint, which results in an increased leaf angle (Zhang et al., 2015). Interestingly, overexpression of *OsIAA1*, a repressor of *OsARF*, also led to increased leaf angle (Song et al., 2009). However, some mutants of transcription factors exhibit reduced leaf angle, such as the *OsLIGULELESS1* (*OsLG1*) mutant, which has a complete absence of the lamina joint, auricle and ligule tissues, resulting in erect leaves (Lee et al., 2007). The reduced leaf angle 1 (RLA1) and BRASSINAZOLE RESISTANT1 (OsBZR1) protein complex is a positive regulator of the BR signaling pathway, and their mutants have an obvious erect leaf phenotype (Bai et al., 2007; Qiao et al., 2017). *INCLINATION1* (*ILI1*) and *ILI1 binding bHLH* (*IBH1*) are direct downstream target genes of the transcription factors *OsBZR1* and *OsLIC*, which are antagonistically involved in regulating the elongation of parenchyma cells in the lamina joint (Zhang L.Y. et al., 2009). It has also been reported that *REGULATOR OF LEAF INCLINATION 1* (*RLI1*) can directly activate the expression of *BRASSINISTEROID UPREGULATED1* (*BU1*) and *BUL1 COMPLEX1* (*BC1*), thereby promoting elongation of lamina joint cells (Ruan et al., 2018). Although multiple types of transcription factors are involved in the regulation of the leaf angle, most of them are related to the metabolism and signaling of plant hormones, such as auxin and brassinosteroids.

In addition to transcription factors, some enzymes related to the transport, metabolism, or signaling of auxin and brassinosteroids are also involved in the development of the lamina joint. For example, *LAZY1* regulates leaf angle by affecting the gravitropism of aboveground tissues of rice (Li et al., 2007), while *Loose Plant Architecture 1* (*LPA1*) regulates the expression of *OsPIN1a* and thus affects the polar transport of auxin (Sun et al., 2019). The *GH3* family gene *LEAF INCLINATION1* (*LC1*) and *an increased number of tillers, enlarged leaf angles, and dwarfism* (*TLD1*) can promote cell elongation by decreasing auxin content at the lamina joint (Zhang S.W. et al., 2009; Zhao et al., 2013). Inhibition of auxin receptor *OsTIR1* expression also resulted in increased leaf angle, suggesting that auxin can negatively regulate leaf angle by inhibiting cell division and cell elongation (Bian et al., 2012). Mutations in the cytochrome *P450* genes *D2, D11* and *OsDWARF*, which are involved in BR biosynthesis (Mori et al., 2002; Hong et al., 2003; Tanabe et al., 2005), and the receptor for BR signaling *OsBRI1* cause shortening of parenchyma cells of the lamina joint and result in an erect leaf phenotype (Yamamuro et al., 2000). The expression and protein stability of the U-type cyclin *CYC U4;1* is negatively regulated by BR, which positively regulates leaf erectness by promoting the division of sclerenchyma cells on the abaxial side of the lamina joint (Sun et al., 2015). From these analyses, it is clear that auxin and BRs are of great importance in the regulation of leaf angle; however, whether other molecules regulate leaf angle by directly or indirectly integrating the metabolism or physiological process of hormones in rice needs to be further evaluated.

In this study, we obtained a mutant *rela (regulator of leaf angle)* with reduced leaf angle in rice by EMS mutagenesis. *RNA-seq* analysis revealed that *OsRELA* is involved in the regulation of *OsEXPA5, OsEXPB6*, and *OsEXPB11* and *OsILI1*, *OsILI4 (OsBU1)*, and *OsILI5 (OsBUL1)* expression. Biochemical and genetic analyses revealed that OsRELA interacts with OsLIC through the conserved C-terminal domain, and the binding of OsRELA can activate the expression of downstream genes repressed by *OsLIC*, thus affecting the leaf angle by positively regulating the expression of *OsILI1* in rice.

## Results

### Phenotypic Analysis of *Rela* Mutant

**FIGURE 1:**
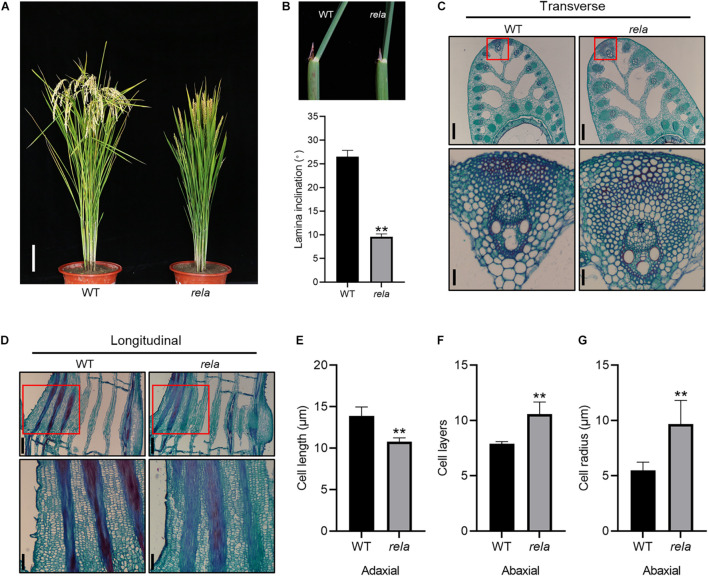
Phenotypic characterization of the *rela* mutant. **(A)** The morphological phenotypes of the wild-type and *rela* at the grain-filling stage. Bar = 20 cm. WT, wild-type. **(B)** Quantification of the leaf inclination of the second lamina joint of wild-type and *rela*. The picture at top shows the second lamina joints. Error bars are SD (*n* = 30). Asterisks indicate significant differences from the WT (**P < 0.01, Student’s *t*-test). **(C)** Transverse section of the lamina joints of wild-type and *rela*. The red box indicates the enlarged abaxial sides of the lamina joint. Bars = 250 μm in the upper panel and 30 μm in the lower panel. **(D)** Longitudinal section of the lamina joints of wild-type and *rela*. The red box indicates the enlarged adaxial sides of the lamina joint. Bars = 250 μm in the upper panel and 30 μm in the lower panel. **(E)** Measurement of lamina joint adaxial cell lengths of wild-type and *rela* [shown in **(D)**]. Error bars are SD (*n* = 60). Asterisks indicate significant differences from the WT (**P < 0.01, Student’s *t*-test). **(F)** The number of sclerenchyma cell layers in the adaxial sides of wild-type and *rela* [shown in **(C)**]. Error bars are SD (*n* = 30). Asterisks indicate significant differences from the WT (**P < 0.01, Student’s *t*-test). **(G)** Measurement of sclerenchyma cell radius in the abaxial sides of wild-type and *rela* [shown in **(C)**]. Error bars are SD (*n* = 30). Asterisks indicate significant differences from the WT (**P < 0.01, Student’s *t*-test).

To screen for genes involved in the regulation of leaf angle in rice, we created a library of *^60^Co-*γ *irradiation-induced mutants* from which we isolated a mutant with semidwarf and leaf erectness (Figures 1A,B), reduced panicle and leaf length phenotypes (Supplementary Figures 1B,C), based on which the mutant was named *regulator of leaf angle* (*rela*). We crossed the *rela* mutant with the wild-type and found that the ratio of wild-type to mutant in the segregating population was 415:134, close to 3:1, indicating that *rela* is a recessive mutation controlled by a single nuclear gene. To further reveal the reason for the reduced leaf angle of the *rela* mutant, we performed a transverse section of the lamina joint and found that the number of sclerenchyma cell layers and the cell area on the abaxial side of the *rela* mutant were increased compared to those of the wild-type (Figures 1C,F,G). Longitudinal section revealed that the length of parenchyma cells on the adaxial side of the *rela* mutant was shorter than that of the wild-type (Figures 1D,E). In addition, longitudinal section of the uppermost internode revealed that the cell length in the *rela* mutant was significantly shorter than that of the wild-type (Supplementary Figures 1A,D–F). These results suggest that *OsRELA* promotes the elongation of cells in the culm and parenchyma cells on the adaxial side and inhibits the division and expansion of sclerenchyma cells on the abaxial side of the lamina joint, thus positively regulating the leaf angle and plant height.

### Cloning of *OsRELA* and Complementation Analysis

**FIGURE 2:**
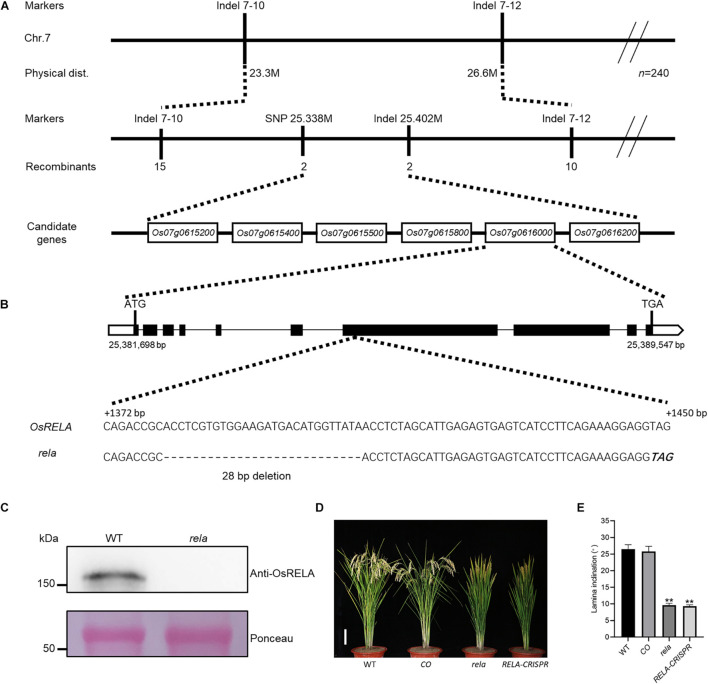
Map-based cloning of *OsRELA* and complementation test. **(A)** Linkage map of the *OsRELA* locus. *OsRELA* is located on the long arm of chromosome 7, between the molecular marker SNP 25.338 M and Indel 25.402 M, a genomic region of ∼64 kb containing six ORFs. The markers, numbers of recombinants and candidate genes are indicated. **(B)** Schematic diagram of the *OsRELA* locus. Comparison with the wild-type sequence revealed a 28-bp deletion (dashed) in the seventh exon, generating a premature stop codon (bold italics). **(C)** Protein levels of OsRELA in the seedlings of wild-type and *rela* detected by immunoblot using anti-OsRELA–specific polyclonal antibodies. Ponceau staining of the Rubisco large subunit is shown as a loading control. Molecular masses of proteins (kDa) are shown on the left. **(D)** Plant morphology of wild-type, *rela/proOsRELA:OsRELA*(*CO*), *rela* and *RELA-CRISPR* at the heading stage. Bar = 10 cm. **(E)** Quantification of the leaf inclination of the second lamina joint of wild-type, *rela/proOsRELA:OsRELA* (*CO*), *rela* and *RELA-CRISPR plants*. Error bars are SD (*n* = 20). Asterisks indicate significant differences from the WT (**P < 0.01, Student’s *t*-test).

To clone the gene encoding *OsRELA*, we crossed the *rela* mutant with *Dular* and obtained a segregating population of the F_2_ generation. By linkage analysis, *OsRELA* was narrowed between two genetic markers, Indel 7–10 and Indel 7–12, located on the long arm of chromosome 7. By further fine mapping using 2871 F_2_ generation plants, we targeted the *OsRELA* gene between SNP 25.338 M, and Indel 25.402 M, and six genes were contained in this 64-kb interval: *Os07g0615200, Os07g0615400, Os07g0615500, Os07g0615800, Os07g0616000, and Os07g0616200* (Figure 2A). Further genome sequencing revealed a 28-bp deletion in the seventh exon of the *Os07g0616000* gene, which shifted the reading frame and eventually led to a premature stop codon, while the rest of the genes were sequenced without mutations (Figure 2B). Our immunoblotting experiments using a specific polyclonal antibody that recognizes the protein encoded by *Os07g0616000* showed that the expression of the protein encoded by *Os07g0616000* was not detected in the *rela* mutant (Figure 2C), indicating that the phenotypes of the *rela* mutant are most likely due to a 28-bp deletion in the *Os07g0616000* gene. Coincidentally, *OsRELA* is located at the same gene locus as the previously reported *DENSE AND ERECT PANICLE 2*. *DEP2* was revealed to be associated with panicle development (Li et al., 2010), and we also found this phenotype in the *rela* mutant; however, the biological function of *OsRELA* involved in the regulation of leaf angle has not been elucidated. Phylogenetic tree analysis revealed that *OsRELA* belongs to a *Spermatophyta*-specific gene family, and its homologs are distributed in *Gymnosperm*, *Amborella*, *Eudicots*, and *Monocots*. However, the gene family has fewer members in *Eudicots*, and there are three copies in Arabidopsis: *AT3G14172, AT1G72410*, and *AT1G17360*. Most of the family members are distributed in the *Monocots*, and there is only one copy in rice (Supplementary Figure 2).

To further clarify the function of *OsRELA* in regulating leaf inclination, we constructed the *pOsRELA:OsRELA* vector and transformed it into the *rela* mutant. The leaf angle of transgenic lines expressing *OsRELA* was restored to the wild-type state (Figure 2D). We also obtained genome-edited lines of *Os07g0616000* using the *clustered regularly interspaced short palindromic repeats* (*CRISPR*)*/CRISPR associated protein 9* (*Cas9*) genome editing tool (Supplementary Figure 3). In all genome-edited homozygous lines obtained, the leaf angle was significantly smaller than that of the wild-type line, consistent with the *rela* mutant phenotype (Figure 2E). These genetic results suggest that *OsRELA* is the key gene that regulates leaf angle in rice.

### Subcellular Localization and Tissue Expression Patterns of *OsRELA*

**FIGURE 3:**
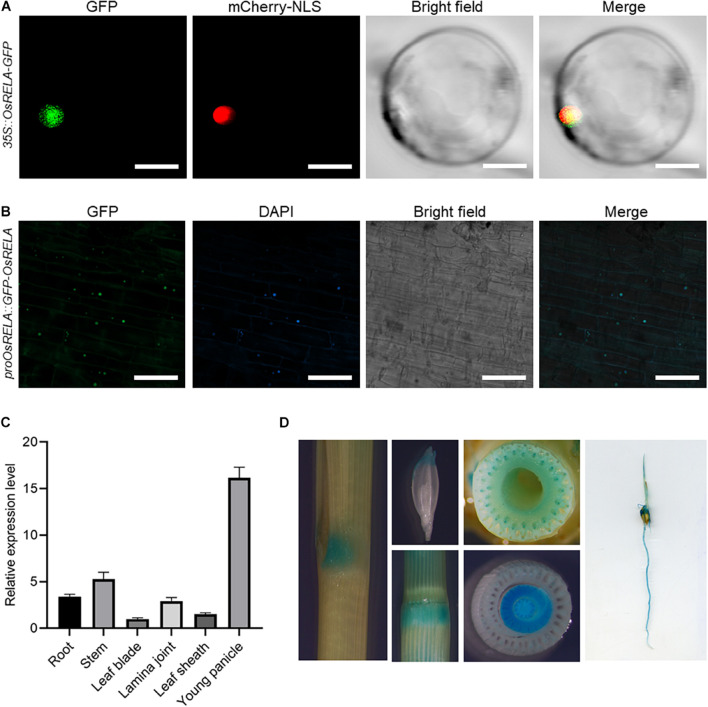
Subcellular localization and tissue expression patterns of *OsRELA*. **(A)** Subcellular localization of the OsRELA-GFP fusion protein in rice protoplasts. mCherry-NLS was used as the nuclear marker. Bar = 20 μm. **(B)** Fluorescent signals in transgenic rice plants expressing GFP-OsRELA. The nuclei were counterstained with 4′,6-diamidino-2-phenylindole (DAPI). Bar = 80 μm. **(C)** RT–qPCR analysis of the expression patterns of *OsRELA* in various tissues, including root, stem, leaf blade, lamina joint, leaf sheath and young panicle. The rice *UBQ* gene was amplified as the internal control. **(D)** Examination of GUS activity in transgenic plants expressing *proOsRELA:GUS*. GUS activity is found in the lamina joint, young florets, stem, vascular bundles, leaf sheath and root.

To determine the localization of *OsRELA* in rice cells, we transformed the *35S:OsRELA-GFP* vector into rice protoplasts for transient expression. Confocal laser scanning microscopy revealed that the fluorescence of the OsRELA-GFP fusion protein merged with the nuclear marker *mCherry-NLS* signal (Figure 3A). Consistently, GFP fluorescence of the GFP-OsRELA fusion protein was colocalized with the nuclear stain DAPI in the *rela/pOsRELA:GFP-OsRELA* lines (Figure 3B), suggesting that OsRELA functions mainly in the nucleus.

To further analyze the expression pattern of *OsRELA*, we performed real-time quantitative PCR (RT–qPCR) to measure the *OsRELA* expression level, and the results showed that *OsRELA* was expressed in various tissues of rice (Figure 3C). GUS staining experiments performed on *pOsRELA:GUS* transgenic plants revealed strong GUS staining in the lamina joint, young florets, stem, vascular bundles, leaf sheath and root (Figure 3D). All these tissues were associated with the phenotypes of the *rela* mutant.

### RNA-seq Analysis of Downstream Genes Regulated by *OsRELA*

**FIGURE 4:**
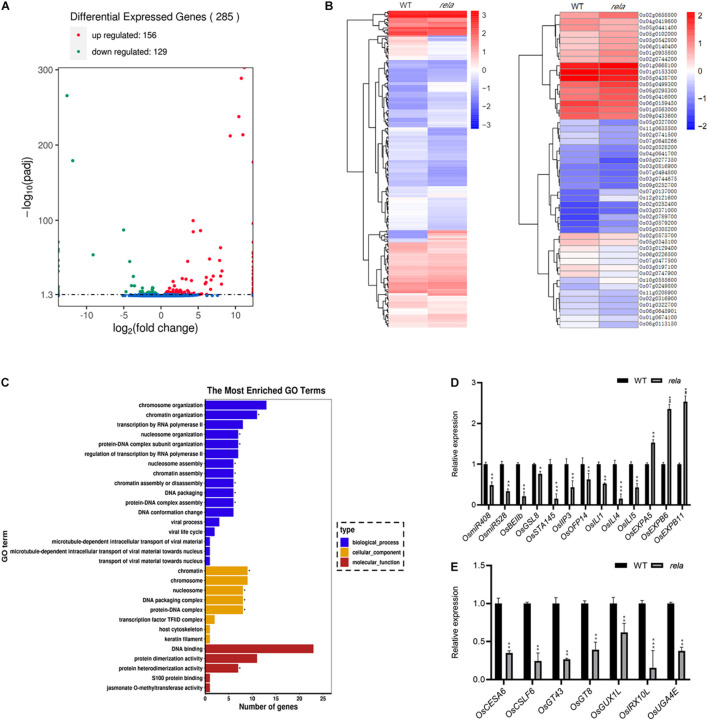
RNA-seq analysis of *OsRELA*-modulated genes. **(A)** A volcano plot illustrating differentially expressed genes from RNA-seq analysis between wild-type and *rela*. Genes upregulated and downregulated are shown in red and green, respectively. Values are presented as the log2 of tag counts. **(B)** Heat map of the RNA-seq analysis results shows all genes that were significantly differentially expressed (left panel) and the representative top 50 genes that were differentially expressed (right panel). **(C)** Gene ontology (GO) functional clustering of all genes that were differentially expressed. **(D)** RT–qPCR validation analysis of the gene expression levels between wild-type and *rela* revealed by RNA-seq. The rice *UBQ* gene was amplified as the internal control. Asterisks indicate significant differences from the WT (*P < 0.05, **P < 0.01, ***P < 0.001, Student’s *t*-test). **(E)** RT–qPCR analysis of the genes involved in cell wall synthesis. The rice *UBQ* gene was amplified as the internal control. Asterisks indicate significant differences from the WT (**P < 0.01, ***P < 0.001, Student’s *t*-test).

To analyze *OsRELA*-regulated genes that participate in the regulation of leaf angle, we performed *RNA-seq* analysis on the *rela* mutant and identified 285 genes with significantly different expression levels compared to the wild-type, including 156 upregulated genes and 129 downregulated genes (Figure 4A). We next performed a hierarchical clustering analysis of all genes that were significantly differentially expressed, and the representative top 50 most significant genes are shown (Figure 4B). In addition, gene ontology (GO) enrichment analysis demonstrated that the molecular functions of the differentially expressed genes were mainly involved in DNA binding (GO:0043566), suggesting that *OsRELA* is likely to be involved in transcriptional regulation by regulating DNA binding (Figure 4C). We then examined the genes with significant expression changes in the wild type and *rela* by RT–qPCR. Consistent with the *RNA-seq* results, the expression of *OsEXPA5, OsEXPB6*, and *OsEXPB11* was significantly upregulated, while the expression of *OsmiR408, OsmiR528, ILA1 interacting protein 3, OsOFP14, OsILI1, OsILI4* (*OsBU1*), and *OsILI5* (*OsBUL1*) was significantly downregulated in the *rela* mutant (Figure 4D), and the expression of seven genes related to cell wall synthesis was also significantly suppressed in *rela* (Figure 4E). It is worth noting that *ILA1 interacting protein 3* (*OsIIP3*), *OsILI1, OsILI4* (*OsBU1*), and *OsILI5* (*OsBUL1*) were reported to positively regulate leaf angle (Tanaka et al., 2009; Zhang L.Y. et al., 2009; Ning et al., 2011; Jang et al., 2017). *Expansin* family genes and cell wall synthesis genes were involved in the process of cell wall loosening and cell extension (Cho and Kende, 1997; Kuluev et al., 2014). The phenotypes of the *rela* mutant are similar to those of BR-deficient and BR-insensitive mutants (Yamamuro et al., 2000; Hong et al., 2003), and both *Expansins* and *ILI* family genes have been reported to be downstream of BR signaling and involved in the regulation of leaf angle (Wang et al., 2018), implying that *OsRELA* may intersect with the BR signaling pathway to regulate leaf inclination in rice.

### OsRELA Physically Interacts With OsLIC and Is Required for OsLIC-Regulated *OsILI1* Expression

**FIGURE 5:**
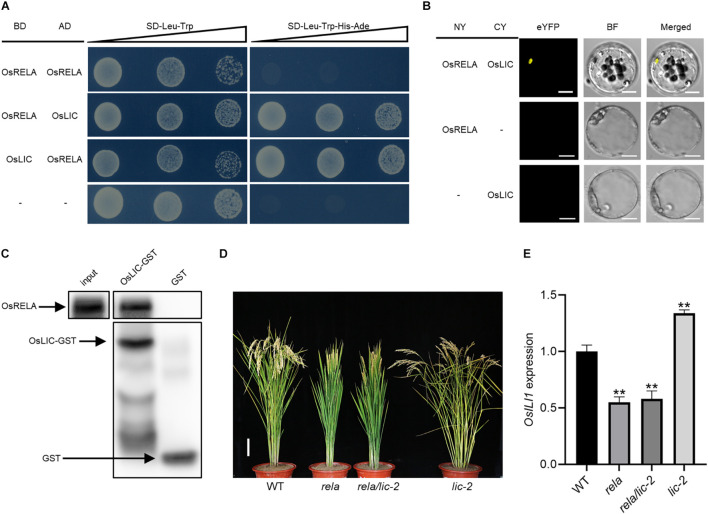
OsRELA physically interacts with and acts downstream of OsLIC. **(A)** Interactions between OsRELA and OsLIC in yeast two-hybrid assays. OsRELA could not form homodimer in yeast. AD, activation domain; BD, binding domain; SD, synthetic dropout; the gradients indicate tenfold serial dilutions. **(B)** BiFC analysis of OsRELA and OsLIC interactions in rice protoplasts. BF, brightfield. Scale bars, 5 μm. **(C)** LIC-GST proteins can pull down OsRELA from total protein extracts of 1-week-old wild-type plants. OsRELA was detected by immunoblotting using anti-OsRELA-specific polyclonal antibodies. **(D)** Plant morphologies of wild-type, *rela, rela/lic-2* and *lic-2* at the heading stage. Bar = 10 cm. **(E)** RT–qPCR analysis of *OsILI1* expression levels in wild-type, *rela, rela/lic-2* and *lic-2* lamina joints. The rice *UBQ* gene was amplified as the internal control. Asterisks indicate significant differences from the WT (**P < 0.01, Student’s *t*-test).

To further reveal the relationship between *OsRELA* and the BR signaling pathway, we obtained *OsLIC* from a rice *cDNA* library by a yeast two-hybrid approach using full-length *OsRELA* as bait (Figure 5A). *OsLIC* negatively regulates leaf angle and acts as an antagonistic transcription factor of OsBZR1 (Zhang et al., 2012). However, OsRELA does not interact with other BR signaling components, such as OsGSK2, DLT, OsBZR1, and RLA1, in yeast (Supplementary Figure 4). Bimolecular fluorescence complementation (BiFC) assays showed that OsRELA and OsLIC interact in rice cells (Figure 5B), and the semi-*in vivo* pull-down assay results also indicated that the OsLIC-GST fusion protein interacts with OsRELA (Figure 5C).

To further clarify the genetic relationship between *OsRELA* and *OsLIC*, using the CRISPR/Cas9 system, a *lic-2* mutant line was obtained by targeting the second exon of the *OsLIC* gene, and DNA sequencing showed that a 1 bp insertion in the second exon of *OsLIC* resulted in premature termination of translation (Supplementary Figure 5). The *lic-2* mutant showed an increased leaf and tiller angle (Wang et al., 2008). We then crossed *rela* with the *lic-2* mutant and found that the phenotypes of the *rela/lic-2* double mutant were similar to those of the *rela* mutant (Figure 5D), implying that the function of *OsLIC* is dependent on *OsRELA*. Quantitative real-time PCR results showed that the expression level of *OsILI1*, the direct downstream target of *OsLIC*, was reduced in both the *rela* and *rela/lic-2* mutants (Figure 5E). The elevated expression level of *OsILI1* and the increased leaf angle in the *lic-2* mutant are consistent with what has been reported previously, suggesting that the regulation of *OsILI1* expression by *OsLIC* is dependent on *OsRELA.*

### The OsRELA-OsLIC Interaction Occurs *via* the Conserved C-Terminal of OsRELA

To clarify the minimal domain of OsRELA that interacts with OsLIC, we performed sequence and bioinformatics analysis on *OsRELA*. We found a speckle-type BTB/POZ domain at the N-terminus of OsRELA by Bioinformatics Toolkit analysis, which is a multifunctional protein–protein interaction motif (Bardwell and Treisman, 1994). Two COILED-COIL domains were predicted by the ExPASy tool kit: 697–724 aa and 798–849 aa. The COILED-COIL domain plays an important role in mediating protein–protein interactions (Burkhard et al., 2001). A nuclear localization sequence was found in its conserved C-terminal domain by SeqNLS software: 1240–1256 aa (Supplementary Figures 6, 7).

**FIGURE 6:**
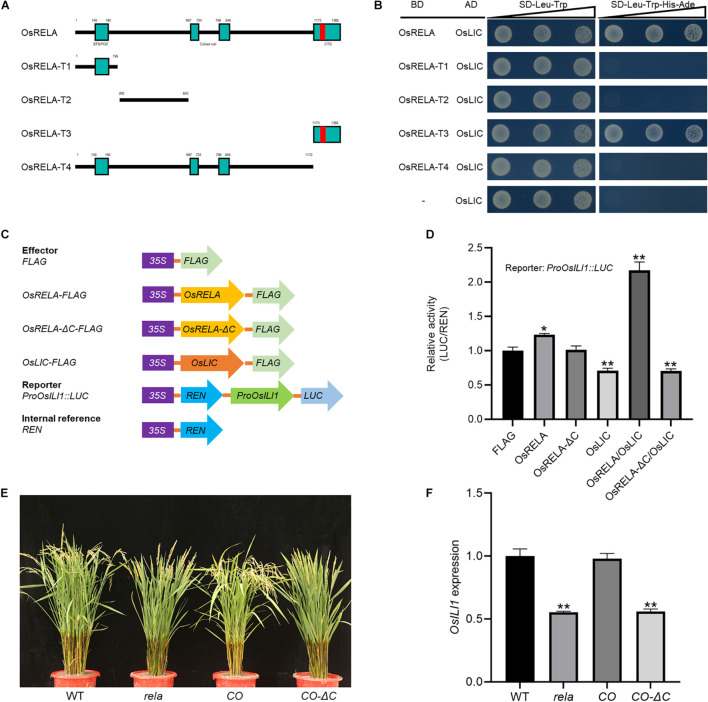
The OsRELA-OsLIC interaction occurs *via* the conserved C-terminal of OsRELA. **(A)** Schematic representation of wild-type and different truncated forms of OsRELA. **(B)** Yeast two-hybrid assays show that OsLIC interacts with OsRELA and OsRELA-T3. AD, activation domain; BD, binding domain; SD, synthetic dropout; the gradients indicate tenfold serial dilutions. **(C)** Schematic diagrams of the reporter and effector constructs. The firefly luciferase (*LUC*) gene driven by the *OsILI1* promoter and the Renilla luciferase (*REN*) reporter gene driven by the *35S* promoter were used as the reporter and internal control, respectively. For the effectors, *OsRELA, OsRELA*Δ*C* and *OsLIC* were fused with *FLAG*. **(D)** Transient gene expression assays in rice protoplasts. The *LUC* reporter gene was cotransfected with *OsRELA, OsRELA*Δ*C* and *OsLIC* or both. Asterisks indicate significant differences from the control (*P < 0.05, **P < 0.01, Student’s *t*-test). **(E)** Plant morphologies of wild-type, *rela, rela/proOsRELA:OsRELA*(*CO*) and *rela/proOsRELA:OsRELA*Δ*C*(*CO–*Δ*C*) at the heading stage. Bar = 10 cm. **(F)** RT–qPCR analysis of *OsILI1* expression levels in wild-type, *rela, rela/proOsRELA:OsRELA*(*CO*) and *rela/proOsRELA:OsRELA*Δ*C*(*CO–*Δ*C*) lamina joints. The rice *UBQ* gene was amplified as the internal control. Asterisks indicate significant differences from the WT (**P < 0.01, Student’s *t*-test).

We next created different truncated forms of *OsRELA* attached to the GAL4-BD domain named BD-OsRELA (1–1365 aa), BD-OsRELA-T1 (1–199 aa), BD-OsRELA-T2 (200–643 aa), BD-OsRELA-T3 (1173–1365 aa) and BD-OsRELA-T4 (1–1172 aa) (Figure 6A). After cotransforming yeast with the OsLIC-AD vector, we found that both OsRELA and OsRELA-T3 interacted strongly with OsLIC, while OsRELA-T1, OsRELA-T2, and OsRELA-T4 did not interact with OsLIC (Figure 6B). The above results suggest that OsRELA interacts with OsLIC through its conserved C-terminal domain. We further used the promoter of *OsILI1* to drive luciferase (*LUC*) as a reporter and *OsRELA-FLAG, OsRELA*Δ*C-FLAG, OsLIC-FLAG*, and *FLAG* as effectors for the transient expression assay (Figure 6C). The results showed that *OsLIC* represses *OsILI1* expression, and when *OsRELA-FLAG* was cotransformed with *OsLIC-FLAG* into rice protoplasts, the expression level of *OsILI1* was elevated. When *OsRELA*Δ*C-FLAG was* cotransformed with *OsLIC-FLAG*, *OsILI1* expression was repressed (Figure 6D). These results indicate that OsRELA interacts with OsLIC through its conserved C-terminal domain and thus abolishes the transcriptional repression of *OsLIC* on *OsILI1.*

Finally, we genetically analyzed the effect of the C-terminal domain of the OsRELA protein on its function and found that deletion of the C-terminal domain could not rescue the phenotypes of the *rela* mutant (Figure 6E). Moreover, quantitative real-time PCR results revealed that the expression level of *OsILI1* was not recovered in the *CO-*Δ*C* lines (Figure 6F), indicating that the regulation of *OsRELA* on *OsILI1* expression through *OsLIC* also depends on its C-terminal domain.

## Discussion

Brassinosteroids are important plant hormones that regulate leaf angle in rice (Tong and Chu, 2018). BR deficiency inhibits the elongation of parenchyma cells on the adaxial side and promotes the division of sclerenchyma cells on the abaxial side of the lamina joint (Zhang L.Y. et al., 2009; Sun et al., 2015); therefore, many BR-deficient and BR-insensitive mutants exhibit an erect leaf phenotype. In this study, we obtained a mutant *rela* with a reduced leaf angle by EMS mutagenesis and found that the cell length in the internode and adaxial parenchyma cells in the lamina joint was reduced compared with that in the wild type. The number of sclerenchyma cell layers and the cell area on the abaxial side were increased, and these phenotypes were similar to those of BR-related mutants. The expression of *OsEXPA5, OsEXPB6*, and *OsEXPB11* was significantly upregulated in the *rela* mutant, while the expression of *OsILI1, OsILI4 (OsBU1), OsILI5 (OsBUL1)*, and genes related to cell wall synthesis were significantly downregulated in the *rela* mutant, and all of these genes were reported to be downstream of the BR signaling pathway. As the only transcription factor in the BR signaling pathway that specifically interacts with OsRELA, OsLIC acts as an antagonistic transcription factor of OsBZR1 to regulate the expression of *OsILI1* and *OsIBH1*, thus affecting leaf inclination. Our results indicate that OsRELA interacts with OsLIC and that OsRELA can act as an important regulator of OsLIC to affect its transcriptional regulation of downstream genes, suggesting that *OsRELA* intersects with the BR signaling pathway by regulating the transcriptional activity of *OsLIC* and is thus involved in the regulation of leaf inclination in rice.

*OsRELA* is located in the same gene locus as previously reported *DENSE AND ERECT PANICLE 2* (*DEP2*) (Li et al., 2010), *ERECT PANICLE2* (*EP2*) (Zhu et al., 2010) and *SMALL AND ROUND SEED1* (*SRS1*) (Abe et al., 2010). *OsRELA/DEP2/SRS1* encodes a protein of unknown function. Through the analysis of the evolutionary relationship between *OsRELA* and its homologs, we found that it is an ancient gene that is conserved among angiosperms, and there are conserved motifs at the N-terminus and C-terminus. Through the *GUS* reporter gene and RT–qPCR results, we found that *OsRELA* is strongly expressed in young tissues, such as young spikelets, florets and lamina joints, and the amino acid sequence at its N-terminus changed when the gene evolved from gymnosperms to angiosperms. The large difference between gymnosperms and angiosperms is that angiosperms produce seeds within an enclosure, suggesting that gene evolution may be related to this. In addition, no functional analysis has been reported for its three homologs in Arabidopsis, and future research should verify whether these three genes are involved in the regulation of Arabidopsis architecture, siliques or seed size by similar mechanisms.

Notably, the N-terminal amino acid sequences of *OsRELA* homologs in Arabidopsis share high similarity to that of COP1-interacting protein 7 (CIP7). It has been reported that CONSTITUTIVE PHOTOMORPHOGENIC1 (COP1) acts as an E3 ubiquitin ligase and is able to inhibit photomorphogenesis in the dark by mediating the ubiquitinated degradation of light-inducible transcription factors, such as *HY5, HYH*, and *AtMYB21* (Holm et al., 2002; Shin et al., 2002; Srivastava et al., 2015). CIP7 is also localized in the nucleus and may act as a transcription factor to activate light-induced gene expression as well as anthocyanin synthesis and chlorophyll accumulation. However, CIP7 RNA-interfering plants do not have defects in light-inhibited hypocotyl elongation (Yamamoto et al., 1998). To further investigate the possible mechanism by which *OsRELA* exerts transcriptional regulatory functions, we performed sequence alignment analysis of *OsRELA* and *CIP7* and found that their N-terminal sequences may encode a speckle-type BTB/POZ domain. The BTB domain (Broad-Complex, Tramtrack, and Bric a brac) is a multifunctional protein–protein interaction domain involved in many biological processes, including transcriptional regulation (Deweindt et al., 1995), ion channel assembly (Aravind and Koonin, 1999) and ubiquitinated protein degradation (Julian et al., 2019). The BTB domain often combines with other structural domains to form multifunctional proteins, BTB-zinc finger proteins were reported to mediate transcriptional repression by interacting with histone deacetylase complexes *N-CoR* or SMRT (Huynh and Bardwell, 1998). *ZBTB24* can specifically bind to the 12-bp DNA motif [CT(G/T)CCAGGACCT] to activate downstream gene expression (Ren et al., 2019), and BACH1 and BACH2 are transcriptional regulators that bind to *Maf* recognition elements to coordinate transcription activation and repression in cooperate with MAFK (Oyake et al., 1996). *LPA1* has also been reported to be involved in a similar process to regulate *PIN1a* expression in rice (Sun et al., 2019). While our results suggest that *OsRELA* functions as a transcriptional activator, whether *OsRELA* has specific DNA binding activity is unclear, and whether its promoter selection for target genes depends on the interacting transcription factors or related proteins needs further validation. In this study, whether the regulation of *ILI* family gene expression by OsRELA requires the binding of the transcription factor OsLIC is unclear. In addition, the specific mechanism of *OsRELA* in regulating gene expression needs to be further evaluated. Whether OsRELA regulates gene expression by changing the three-dimensional conformation of chromosomes similar to that of chromatin remodeling factors or regulates the activation or repression of gene expression by recruiting histone modification complexes needs to be further evaluated. Further studies are needed to reveal the specific mechanism of *OsRELA*.

**FIGURE 7:**
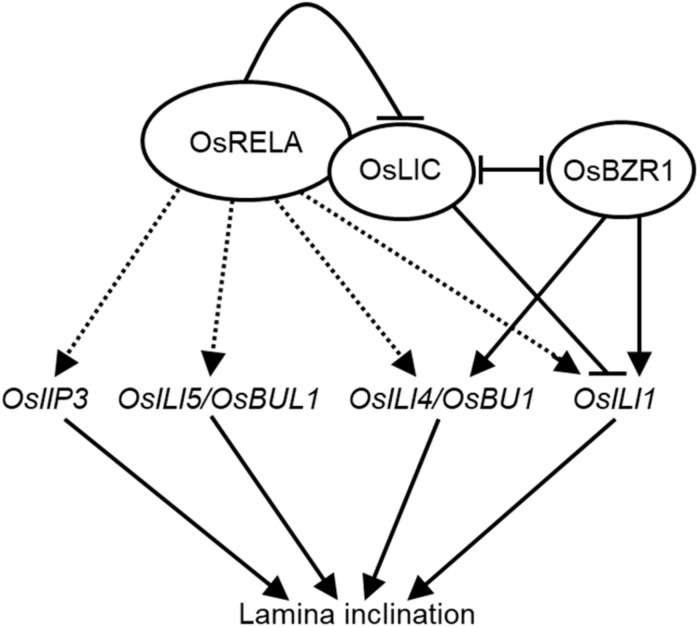
A model for OsRELA in regulating the lamina inclination. OsRELA positively regulates the expression of *OsIIP3, OsILI5/OsBUL1, OsILI4/OsBU1*, and *OsILI1*, which are positive regulators involved in the lamina inclination. Since it has been reported that OsBZR1 and *OsLIC* are pair of antagonistic transcription factors that involved in the lamina inclination by regulate the expression of *OsILI1*. The binding of OsRELA can activate the expression of *OsILI1* repressed by OsLIC as well. Whether the OsRELA directly or indirectly activates the listed gene expressions (dashed arrows) should be elucidated in the future.

In this study, we identified *OsRELA* through forward genetics, which is an important regulator of leaf angle in rice. As summarized in Figure 7, OsRELA regulates several lamina inclination-related gene expressions, which have been demonstrated as the positive regulators in rice leaf inclination (Tong and Chu, 2018). Furthermore, OsRELA affects leaf angle by influencing the transcriptional regulation of *OsILI1 via* interacting and inhibiting OsLIC, which antagonizes with OsBZR1 each other (Zhang et al., 2012), the key transcriptional factor in BR signaling. However, OsRELA does not interact with OsBZR1, indicating they response to different environmental or developmental cues to regulate lamina inclination *via* suppressing the OsLIC in rice. Collectively, our data not only clarify a new mechanism of leaf inclination regulation but also provide a reference and new genetic resources for future crop improvement.

## Materials and Methods

### Plant Materials and Growth Conditions

In this study, Nipponbare *rice* (*Oryza sativa subsp. japonica*) and *Dular rice* (*Oryza sativa L. ssp. indica*) were used as wild-type controls, and the *rela* mutant was obtained from EMS-induced mutant populations. The F_2_ segregation population used for genetic segregation analysis was obtained by crossing the *rela* mutant with *Nipponbare*. The F_2_ segregant population used for map-based cloning was obtained by crossing the *rela* mutant with *Dular*. The rice materials used in this study were grown in the fields of the Chinese Academy of Agricultural Sciences under natural conditions.

### Paraffin Section Analysis

After the mature lamina joints of the wild-type and *rela* mutant were separated, they were rapidly fixed in *formaldehyde-acetic acid-alcohol* (*FAA*) at 4°C for 72 h. Subsequently, the samples were dehydrated through a gradient ethanol solution. The dehydrated samples were subjected to a xylene:ethanol clear solution of 1:2, 1:1, and 2:1 and finally transitioned to xylene. The samples were then incubated in a xylene:Paraplast Plus solution (Sigma–Aldrich) solution of 1:1 at 37°C for 48 h. The samples were embedded in Paraplast Plus for 3 days. The samples were then sectioned into 8-μm-thick sections by an RM2245 rotary microtome (Leica). After the removal of Paraplast Plus with xylene:ethanol ratios of 2:1, 1:1, and 1:2, the sections were rehydrated with gradient ethanol, followed by safranin-fixed green staining. Images were taken with an ICC50 HD microscope (Leica), and the cell length and cell area were subsequently measured using ImageJ.

### Map-Based Cloning of *rela*

A total of 154 primer pairs exhibiting polymorphisms between *Nipponbare* and *Dular* were identified from our primer library. We targeted *OsRELA* between two genetic markers, Indel 7–10 and Indel 7–12, located on the long arm of chromosome 7. By further fine-mapping using the F_2_ generation population of 2871 progeny, we targeted the *OsRELA* gene between two newly developed genetic markers. We then performed PCR amplification and sequencing of the genomic sequences of all genes within this 64-kb interval in wild-type and *rela* mutants.

### Vector Construction and Plant Transformation

For complementation assays, the putative 2.2-kb promoter region of *OsRELA* was amplified using the OsRELA-Pro primer pairs, and the coding region was amplified using the OsRELA-CDS and OsRELA-CDSΔC primers and inserted into the pCAMBIA1300 binary vector using *Eco*RI/*Hin*dIII restriction sites.

To knock out the *OsRELA* and *OsLIC* genes, we selected 20-bp gene-specific spacer sequences in the coding region and cloned them into the sgRNA-Cas9 vector to obtain *OsRELA-CRISPR* and *OsLIC-CRISPR* vectors.

To obtain *OsRELA* overexpression lines, the promoter of *OsRELA* was amplified by primer OsRELA-Pro, and the full-length CDS was amplified by using primer OsRELA-GFP and inserted into the pCAMBIA1300 binary vector using *Eco*RI/*Hin*dIII restriction sites to obtain *proOsRELA:GFP-OsRELA.*

To determine the expression pattern of *OsRELA*, a 2.2-kb region upstream of the ATG start codon was amplified using OsRELA-GUS primers, and the PCR product was cloned into the pCAMBIA1391z binary vector using *Hin*dIII/*Eco*RI restriction sites to obtain the *proOsRELA:GUS* vector.

The above vectors were subsequently transformed into wild-type and *rela* mutant *via* Agrobacterium-mediated transformation.

### Preparation and Determination of OsRELA Specific Antibodies

An *in vitro* purified OsRELA polypeptide (*Os07g0616000*, 500–740 aa) fragment was injected into rabbits to generate the corresponding polyclonal antibody at ABclonal Technology. The specificity of the polyclonal antibody against OsRELA was subsequently verified by immunoblotting assays using wild-type rice seedlings. Samples were well ground in liquid nitrogen and then incubated with protein extraction buffer (50 mM Tris-HCl at pH 7.5, 150 mM NaCl, 10 mM MgCl_2_, 1 mM EDTA, 10% glycerol, and protease inhibitor cocktail) at 4°C for 15 min. After centrifugation at 4°C for 10 min, the supernatant was boiled for 10 min and separated on a 6% SDS–PAGE gel. The anti-OsRELA polyclonal antibody was diluted at a concentration of 1:2000 and detected by chemiluminescence reagent (GE Health care), and the Rubisco large subunit was used as the endogenous control.

### Subcellular Localization and GUS Activity Measurements

To determine the subcellular localization of the OsRELA protein, the 4095-bp coding region was cloned into the pAN580 vector using the *Xba*I restriction site. The *35S:OsRELA-GFP* vector was subsequently introduced into rice protoplasts as previously described (Zhang et al., 2011), and the fluorescence of GFP was visualized using a confocal laser scanning microscope (LSM 980; Zeiss).

Different tissues of *proOsRELA:GUS* transgenic plants were incubated in GUS staining buffer (1 M sodium phosphate, pH 7.0, 0.5 M EDTA, pH 8.0, 10% Triton X-100, 50 mM K3Fe(CN)_6_, 0.1 M X-Gluc) at 37°C after isolation. After 12–16 h, the samples were decolorized using 75% ethanol. The pictures were photographed using an ICC50 HD microscope (Leica).

### Bioinformatics Analyses

The sequences of *OsRELA* and *OsLIC* were obtained from The Rice Annotation Project^1^. Homologous of *OsRELA* in plants were obtained from the NCBI BLAST server^2^. Multiple sequence alignment was performed using DNAMAN9 with default settings. A neighbor-joining tree of *OsRELA* and its homologs was generated by MEGA7 with 1000 bootstrap replicates. The protein structure analysis was performed using the following software:

HHpred: https://toolkit.tuebingen.mpg.de/tools/hhpred

COILS: https://embnet.vital-it.ch/software/COILS_form.html

SeqNLS: http://mleg.cse.sc.edu/seqNLS/

### RNA Extraction, Real-Time Quantitative PCR, and RNA-seq Analyses

Total RNA was extracted from lamina joints of rice seedlings using TRIzol reagent (Invitrogen). First-strand cDNA synthesis was performed using the PrimeScript 1st strand cDNA Synthesis Kit (6110A). RT–qPCR was performed as previously described (Zhou et al., 2008), and the rice *ubiquitin* gene was used as an internal control.

The differential gene expression analysis of 2-week-old rice seedlings of wild-type and *rela* mutant were performed by Allwegene Technology Inc^3^.

### Yeast Two-Hybrid Assays

The full-length coding region of *OsRELA* was cloned into the pGBKT7 vector as bait, and then yeast two-hybrid screening was performed with a rice cDNA library. The positive clones were subsequently identified by sequencing. The full-length coding regions of *OsRELA, OsLIC, OsBZR1, RLA1, DLT*, and *OsGSK2* were cloned into the corresponding pGBKT7 and pGADT7 vectors using *Eco*RI/*Bam*HI restriction sites. The corresponding bait and prey constructs were cotransformed into the yeast strain AH109 (Clontech) and grown on the selective medium SD-Trp/-Leu. The positive clones were then transferred to the selective medium SD-Trp/-Leu/-His/-Ade.

### Bimolecular Fluorescence Complementation Assays

The full-length coding region of *OsRELA* was amplified and cloned into the *pSPYNE* vector using *Eco*RI/*Sal*I to obtain *NY-OsRELA*, and the full-length coding region of *OsLIC* was amplified and cloned into the *pSPYCE* vector using *Eco*RI/*Sal*I to obtain *OsLIC-CY*. *OsRELA-NY/OsLIC-CY, OsRELA-NY/pSPYCE*, and *pSPYNE/OsLIC-CY* were cotransformed into rice protoplasts by PEG-mediated transformation. The fluorescence of YFP was visualized using a confocal laser scanning microscope (LSM 980; Zeiss).

### Semi-*in Vivo* Pull-Down Assay

To test the interaction between OsRELA and OsLIC, the full-length coding region of OsLIC was amplified and cloned into pGEX-4T-1 using *Eco*RI/*Xho*I, and the vector was subsequently transformed into *E. coli* strain BL21 to obtain the OsLIC-GST fusion protein. Two-week-old wild-type seedlings were ground in liquid nitrogen and solubilized with 2X protein extraction buffer [100 mM Tris-HCl, pH 7.5, 300 mM NaCl, 2 mM EDTA, pH 8.0, 1% TrionX-100, 10% (v/v) glycerol, and protease inhibitor mixtures (M307; AMRESCO)]. The semi-*in vivo* pull-down assay was performed as previously described (Qiao et al., 2017).

### Transient Expression Assay in Rice Protoplasts

In the dual-luciferase assay, the 2.2-kb promoter region of *OsILI1* was cloned upstream of the firefly luciferase gene (*LUC*) in pGreenII 0800-LUC to generate the *OsILI1-LUC* reporter vector. *OsLIC-FLAG, OsRELA-FLAG, OsRELA*Δ*C-FLAG*, and *FLAG* were cloned into pGreenII 62-SK as effectors using *Xba*I/*Hin*dIII restriction sites. The combined reporter and effector vectors were cotransformed into rice protoplasts by PEG-mediated transformation. *Renilla reniformis* (*Ren*) driven by the 35S promoter was used as an internal control. The LUC activity was quantified with a Dual-Luciferase Assay Kit (Promega) following the manufacturer’s recommendations, and the relative LUC activity was calculated as the ratio of LUC/Ren.

## Accession Numbers

Sequence data from this article can be found in the database of The Rice Annotation Project (RAP) under the following accession numbers: *OsRELA*,Os07g0616000;*OsLIC*,Os06g0704300;*OsmiR408*,Os01g0322700;*OsmiR528*,Os03g0129400;*OsBEIIb*,Os02g0528200;*OsG SL8*,Os06g0113150;*OsSTA145*,Os05g0327000;*OsIIP3*,Os02g0575700;*OsOFP14*,Os05g0441400;*OsILI1*,Os04g0641700;*OsILI4*(*OsB U1*),Os06g0226500;*OsILI5*(*OsBUL1*),Os02g0747900;*OsEXPA5*, Os02g0744200;*OsEXPB6*,Os10g0555600;*OsEXPB11*,Os02g0658800;*OsCESA6*,Os07g0252400;*OsCSLF6*,Os08g0160500;*OsGT43*,Os04g0650300;*OsGT8*,Os02g0739400;*OsGUX1L*,Os01g0880200;*OsI RX10L*,Os01g0926700;*OsUGA4E*,Os08g0526100 and *UBIQUI TIN*, Os03g0234200.

## Primer Sequences

The primers used are shown in Supplementary Table 1 and Supplementary Table 2.

## Data Availability Statement

The datasets presented in this study can be found in online repositories. The names of the repository/repositories and accession number(s) can be found in the article/Supplementary Material.

## Author Contributions

T-gL conceived the project and designed the research. Z-gZ and P-fA conceived the original screening, constructed the mapping population. C-lZ and BX carried out most of the experiments, map-based cloning of *OsRELA* gene, paraffin section, genetic analysis, and chemical parameters. CD completed the protein purification. Z-yS and S-zT performed rice transformation. S-wZ and Z-gZ designed the experiments, analyzed, and wrote the data with C-lZ. All authors contributed to the article and approved the submitted version.

## Conflict of Interest

The authors declare that the research was conducted in the absence of any commercial or financial relationships that could be construed as a potential conflict of interest.

## Publisher’s Note

All claims expressed in this article are solely those of the authors and do not necessarily represent those of their affiliated organizations, or those of the publisher, the editors and the reviewers. Any product that may be evaluated in this article, or claim that may be made by its manufacturer, is not guaranteed or endorsed by the publisher.

## Footnotes

1
https://rapdb.dna.affrc.go.jp

2
http://blast.ncbi.nlm.nih.gov/Blast.cgi

3
http://www.allwegene.com/
