# Supplementary material for: OsRELA Regulates Leaf Inclination by Repressing the Transcriptional Activity of OsLIC in Rice
Source: Front Plant Sci. 2021 Oct 1;12:760041. doi: 10.3389/fpls.2021.760041 (PMC8519309; doi:10.3389/fpls.2021.760041)
Supplement: Supplementary file 1 [file Data_Sheet_1.PDF]

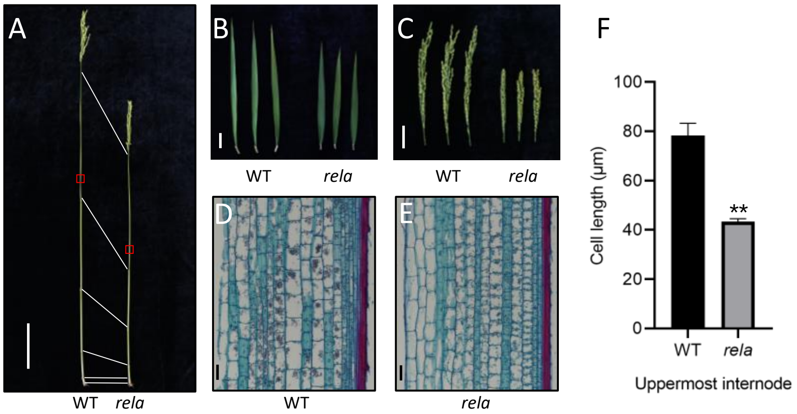


**Supplemental Figure 1.** Comparisons of culm, leaf and panicle length between wild-type and *rela* plants at the mature stage.

**(A)** The culm length of wild-type and *rela*. Bars= 15 cm.

**(B)** Phenotype of flag leaves of wild-type and *rela*. Bars= 3 cm.

**(C)** Phenotype of panicles of wild-type and *rela*. Bars= 5 cm.

**(D)** and **(E)** Longitudinal section of the uppermost internode indicated by the red box in **[A]** of wild-type and *rela*. Bars= 50μm.

**(F)** Measurement of the uppermost internode cell lengths of wild-type and *rela* (shown in **[D]** and **[E]**). Error bars are SD (n= 30). Student's *t* test: **P < 0.01.

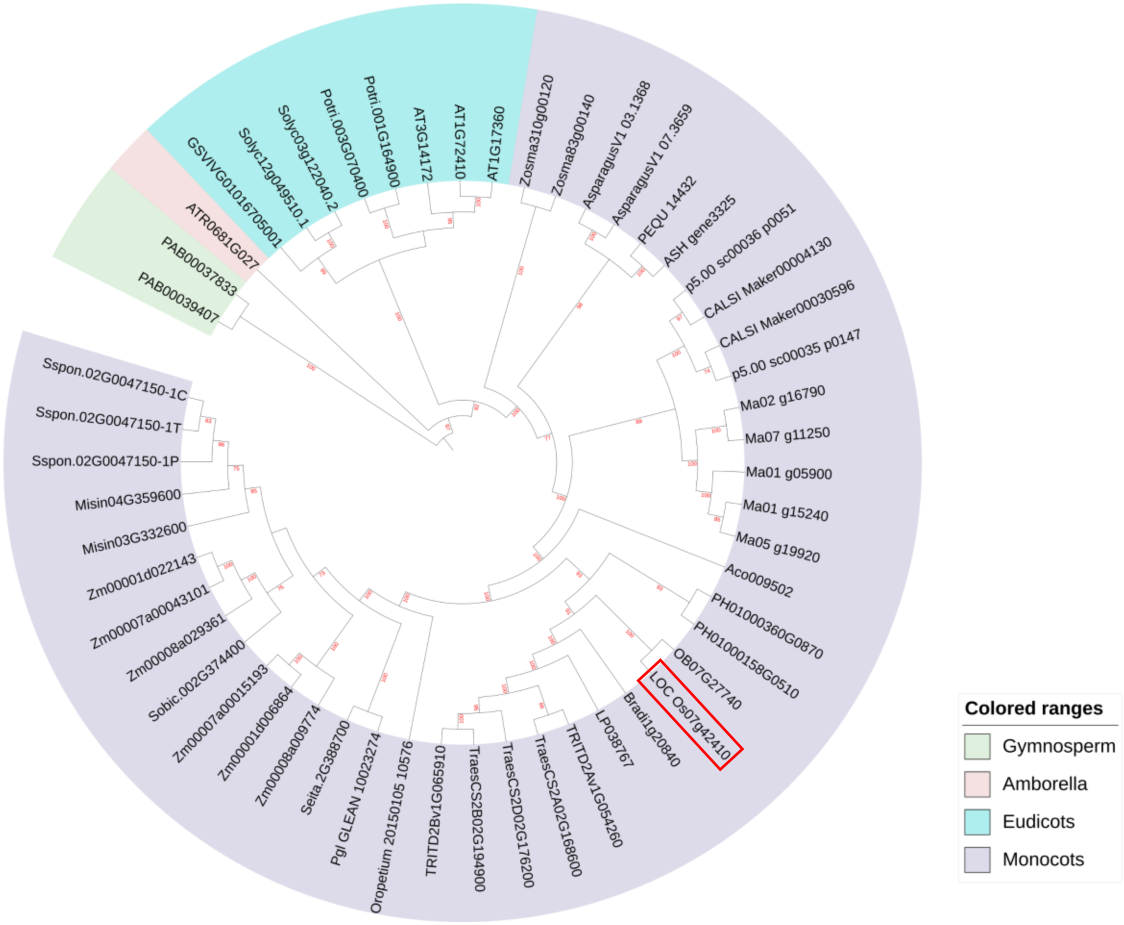


**Supplemental Figure 2.** Phylogenetic tree of OsRELA and its homologs in plants.
The neighbor-joining tree was created using the MEGA 7.0 program with the p-distance model, bootstrap analysis was performed with 1000 replicates, and numbers in branches indicate bootstrap values (percent). *Gymnosperms* are highlighted in green, *Amborella* are highlighted in pink, *Eudicots* are highlighted in blue and *Monocots* are highlighted in gray.

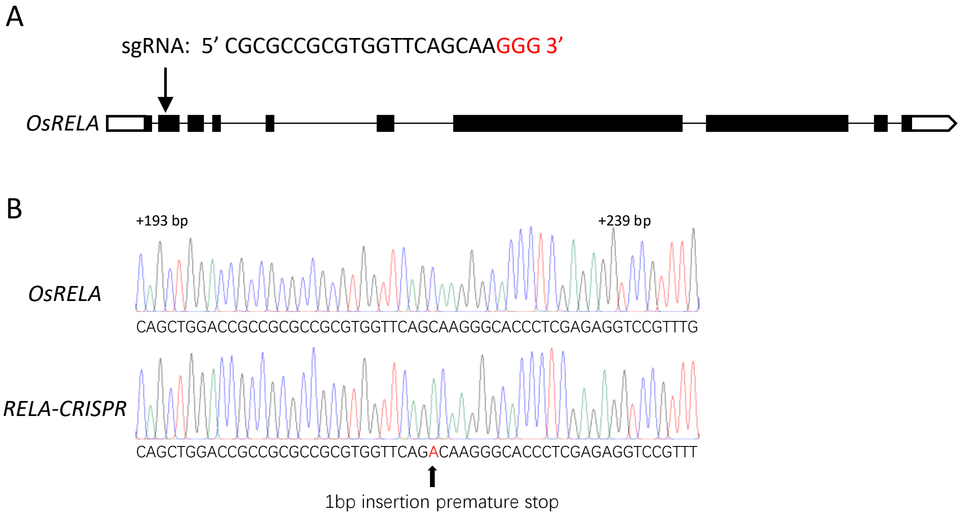


**Supplemental Figure 3.** Generation and identification of *OsRELA* mutants generated by CRISPR/Cas9.
**(A)** The gene-specific spacer sequence was selected in the second exon of *OsRELA*.
**(B)** Chromatograms of the *OsRELA* sequence in wild-type and *RELA-CRISPR*. Insertion of 1-bp was indicated by the red letter.

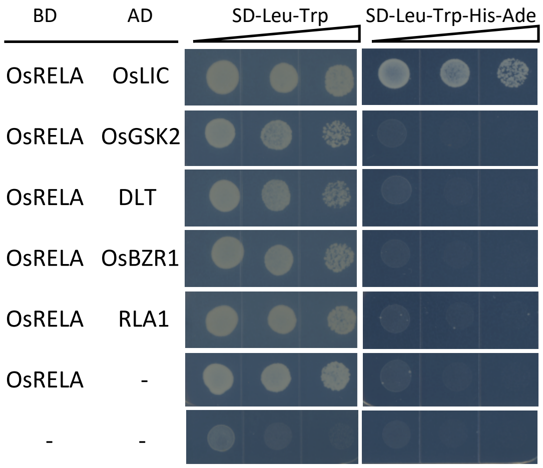


**Supplemental Figure 4.** OsRELA does not interact with OsGSK2, DLT, OsBZR1 or RLA1 in yeast. AD, activation domain; BD, binding domain; SD, synthetic dropout; the gradients indicate tenfold serial dilutions.

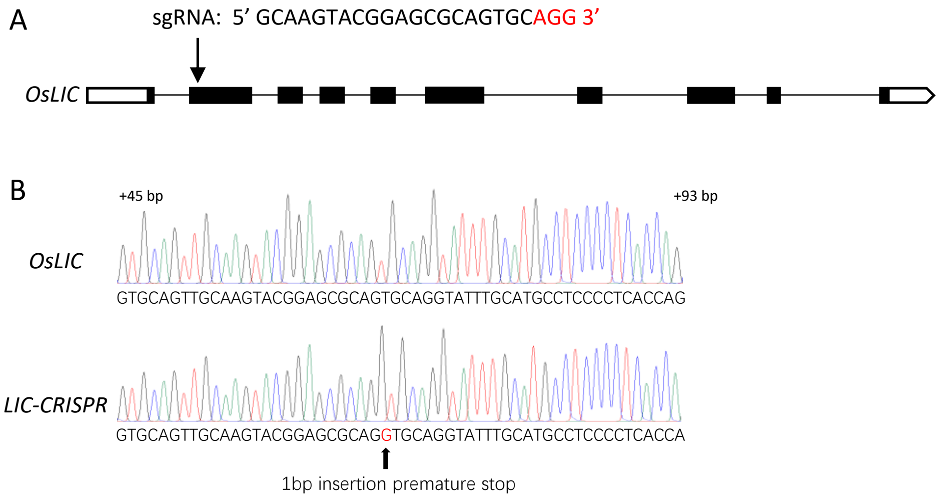


**Supplemental Figure 5.** Generation and identification of *OsLIC* mutants generated by CRISPR/Cas9.
**(A)** The gene-specific spacer sequence was selected in the second exon of *OsLIC*.
**(B)** Chromatograms of the *OsLIC* sequence in wild-type and *LIC-CRISPR*. Insertion of 1-bp was indicated by the red letter.

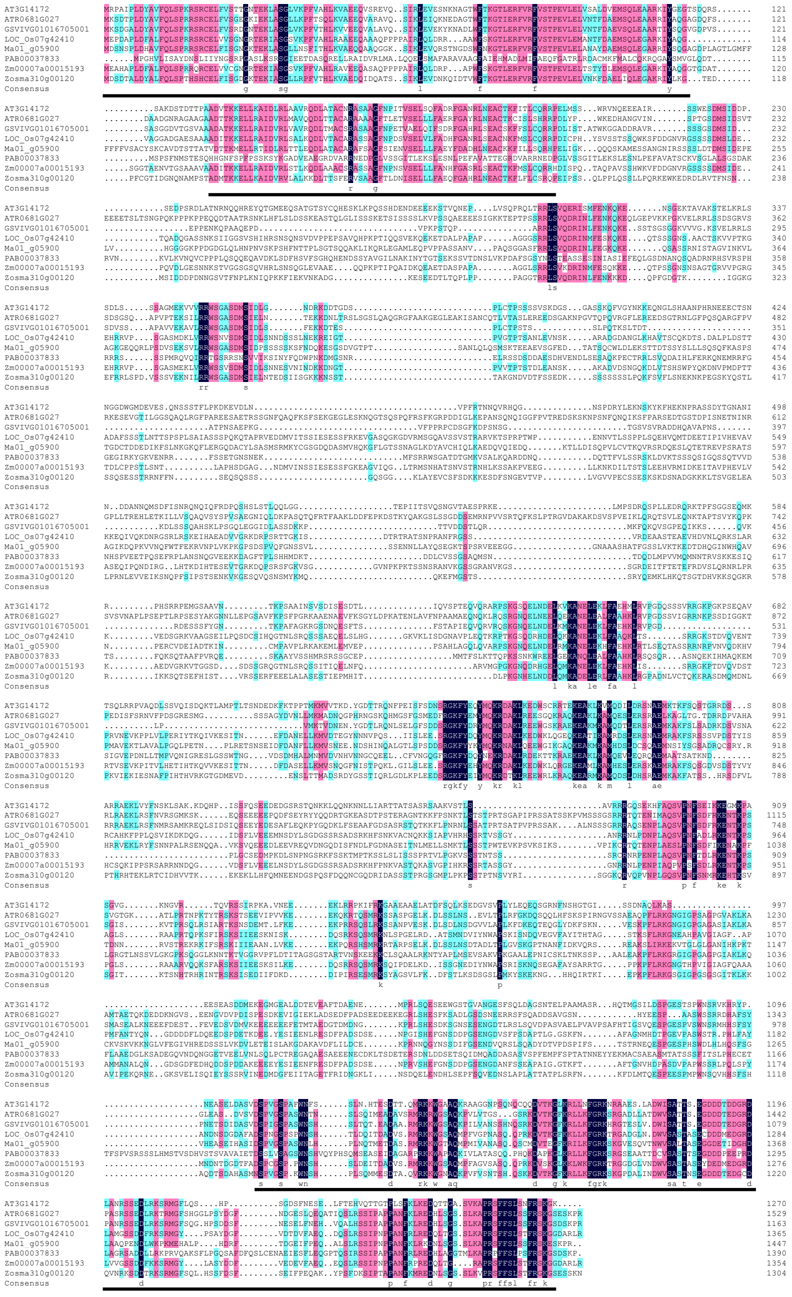

**Supplemental Figure 6.** Characterization and Sequence Analysis of OsRELA.
Amino acid sequence alignment of sequences from rice and other selected species (*Arabidopsis thaliana, Amborella trichopoda, Vitis vinifera, Musa acuminata, Picea abies, Zea mays and Zostera marina*) was conducted by DNAMAN Version 9 software. Identical and similar residues are displayed in black and pink, respectively. The conserved C-terminal and N-terminal domains are underlined.

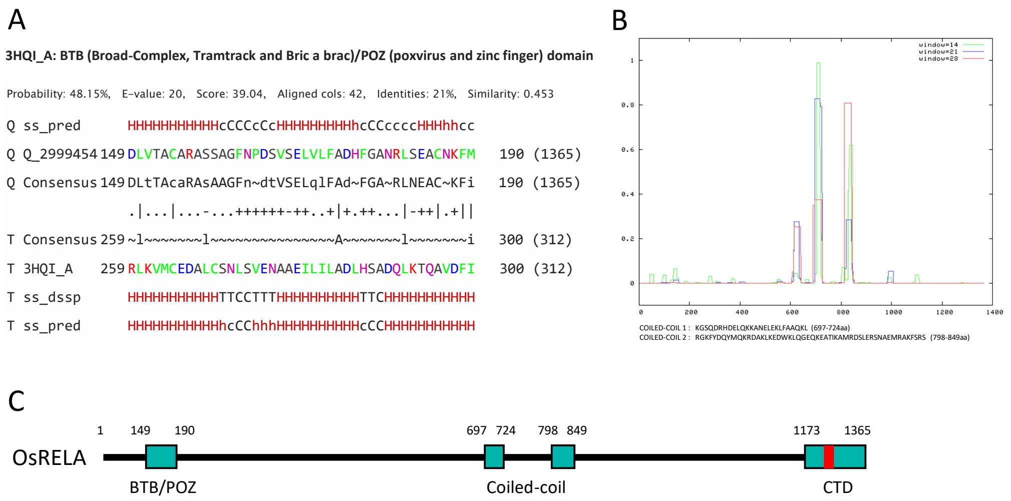


**Supplemental Figure 7.** *OsRELA* encodes a BTB/POZ and coiled-coil domain protein.

**(A)** Alignment of the BTB/POZ domain between OsRELA and human SPOP. Full-length OsRELA protein was blasted against the 'PDB_mmCIF70_13_Jul' database using HHpred of the MPI Bioinformatics Toolkit (https://toolkit.tuebingen.mpg.de/tools/hhpred).

**(B)** The prediction of coiled-coil regions in OsRELA by COILS (https://embnet.vital-it.ch/software/COILS_form.html).

**(C)** Domain schematic of OsRELA protein and the red box in the CTD domain indicate the nuclear localization signal predicted by SeqNLS (http://mleg.cse.sc.edu/seqNLS/).
