# Supplementary material for: OsRELA Regulates Leaf Inclination by Repressing the Transcriptional Activity of OsLIC in Rice
Source: Front Plant Sci. 2021 Oct 1;12:760041. doi: 10.3389/fpls.2021.760041 (PMC8519309; doi:10.3389/fpls.2021.760041)
Supplement: Supplementary file 2 [file Data_Sheet_2.PDF]

Supplemental Table S1. Primers Used for Map-based Cloning.

Primers	Sequences
Indel 7-10-F	5' GCCAGATCGGAAGACTTGATCT 3'
Indel 7-10-R	5’ TGACGATAGGTGTGACGACCT 3’
Indel 25.321-F	5’ AGGGGAAGACCATCTGGAAG 3’
Indel 25.321-R	5' CAGCGAGGTGAAGATCCAGT 3'
Indel 25.335-F	5' GATTTGCAGGAGGCTTTCAG 3'
Indel 25.335-R	5’ CAGCTTGGCATCTGGTGTAA 3’
SNP 25.338-F	5’ GCAGCAGCATTAGCAGTGAG 3’
SNP 25.338-R	5’ GGGAAGGAAGGAGAGAGTGG 3’
Indel 25.402-F	5’ GAGAGAGGGTTGGGGAGTTTA 3’
Indel 25.402-R	5’ CAAAATTGTGCGCGAAAAC 3’
Indel 25.407-F	5’ TCAATGCGAGTGATCACCAT 3’
Indel 25.407-R	5’ AGGTCAATTCAGCCACTTCG 3’
Indel 7-12-F	5’ CACGGATGTAGTAAACTAGGAT 3’
Indel 7-12-R	5’ GCCTGAAACTGTTCTAGAAGT 3’
