# Supplementary material for: OsRELA Regulates Leaf Inclination by Repressing the Transcriptional Activity of OsLIC in Rice
Source: Front Plant Sci. 2021 Oct 1;12:760041. doi: 10.3389/fpls.2021.760041 (PMC8519309; doi:10.3389/fpls.2021.760041)
Supplement: Supplementary file 3 [file Data_Sheet_3.PDF]

Supplemental Table S2. Primers Used for RT-qPCR Analysis.

Primers	Sequences
OsmiR408-F	5' TGCAGCTGCACTGCCTCTTC 3'
OsmiR408-R	5’ GTCGTATCCAGTGCAGGGTCCGAGGTATTCGCACTGGATACGACGCCAGG 3’
OsmiR528-F	5’ CTGCTCCAGGTCTGAATTCATA 3’
OsmiR528-R	5' GTCCATTTCGTTGTCCTGAATC 3'
OsBEIIb-F	5' GAGCAAGAAAAACTAGTTGCCA 3'
OsBEIIb-R	5’ AAGCAAGCTGTCTAGTCTATCC 3’
OsGSL8-F	5’ GCGAATCGGGAACTTTTAGTAC 3’
OsGSL8-R	5’ AGTCTATCGGATGGTGATTGTC 3’
OsSTA145-F	5’ AAAGAGGGAAAATGAAAAGCCG 3’
OsSTA145-R	5’ CAATTGCAAACCCATCACAATG 3’
OsIIP3-F	5’ ATCTGATGGATAATTCCGCAGT 3’
OsIIP3-R	5’ TGCCATGGATACTACTGTTCTC 3’
OsOFP14-F	5’ GTCCTCTACACCATCAACTCG 3’
OsOFP14-R	5’ GAACGGAAGGAATGAGCTCTAG 3’
OsILI1-F	5’ TGCTGAACAGAACTGGAGTATT 3’
OsILI1-R	5’ GGCACAGATATATCGCTTCCTA 3’
OsILI4-F	5’ ACTTCACTTCAGTTCAGTAGGG 3’
OsILI4-R	5’ ATGAGATCAAGCTGGCTACTAC 3’
OsILI5-F	5’ AAATCAACGAGCTCATCTCCAA 3’
OsILI5-R	5’ GTGAAGGCTCTTGATGTAGTTG 3’
OsEXPA5-F	5’ TGGTGGTAAGGCTGTGGCTTGA 3’
OsEXPA5-R	5’ CGGTACAGACAACAACCAGGCA 3’
OsEXPB6-F	5’ AATTTGCGTGGGATTGAGGTGT 3’
OsEXPB6-R	5’ TGGGTAGTACAGTGACAGTGGG 3’
OsEXPB11-F	5’ TGCAGTGCAGAGTTGCGGTAA 3’
OsEXPB11-R	5’ CAGAGACCGTGGAGGGAAGAAC 3’
OsCESA6-F	5’ GAGCTTACTTGGTGGAAG 3’
OsCESA6-R	5’ AATAAACAATTGGCATCTTC 3’
OsCSLF6-F	5’ GGTGTGGTGTATGATTGT 3’
OsCSLF6-R	5’ CAGCCTCTCTTTCTCTCA 3’
OsGT43-F	5’ CACACGAATGGAAAATTG 3’
OsGT43-R	5’ ACCTTCAGTATTCAATAGC 3’
OsGT8-F	5’ AGATTGATTCGTTAGGCTTC 3’
OsGT8-R	5’ AAATGAAACCTTTATCCTATCCA 3’
OsGUX1L-F	5’ ACTGTAACTGGAATAACC 3’
OsGUX1L-R	5’ AAGACAATAGTGCTGAAG 3’
OsIRX10L-F	5’ GCAGGCTCATCTTATTCC 3’
OsIRX10L-R	5’ TGCTGGTATCGTAGAACA 3’
OsUGA4E-F	5’ ATTGTCCTTGGAGGCTAG 3’

OsUGA4E-R	5’ AATTCAGCATGGTTGATTG 3’
UBQ-F	5’ AACCAGCTGAGGCCCAAGA 3’
UBQ-R	5’ ACGATTGATTTAACCAGTCCATGA 3’
